# Strategies Using Bio-Layer Interferometry Biosensor Technology for Vaccine Research and Development

**DOI:** 10.3390/bios7040049

**Published:** 2017-10-31

**Authors:** Rejane L. Petersen

**Affiliations:** Pall Fortebio, 47661 Fremont Boulevard, Fremont, CA 94538, USA; rejane_petersen@pall.com

**Keywords:** BLI, vaccine, biosensor, label-free, real-time

## Abstract

Bio-layer interferometry (BLI) real-time, label-free technology has greatly contributed to advances in vaccine research and development. BLI Octet platforms offer high-throughput, ease of use, reliability, and high precision analysis when compared with common labeling techniques. Many different strategies have been used to immobilize the pathogen or host molecules on BLI biosensors for real-time kinetics and affinity analysis, quantification, or high-throughput titer. These strategies can be used in multiple applications and shed light onto the structural and functional aspects molecules play during pathogen-host interactions. They also provide crucial information on how to achieve protection. This review summarizes some key BLI strategies used in human vaccine research and development.

## 1. Introduction

Real-time label-free (RT-LF) technologies have greatly contributed to the research, development, and production of vaccines in recent years. Biosensor technologies based on Surface Plasmon Resonance (SPR), Surface Plasmon Resonance Imaging (SPRi), and Bio-Layer Interferometry (BLI) have proven the practicality and effectiveness of monitoring molecular interactions, as binding events can be monitored in real-time without the requirement of supplementary and costly labeling. These RT-LF biosensor techniques are very powerful and useful for the characterization of molecular interactions during most stages of vaccine production and development. Extensive information is available for multiple platforms of SPR [1]. This review article focuses on the uses of BLI technology and its diversity of platforms used at various stages of vaccine research and development.

The use of the BLI technology has increased rapidly in the past decade, and this trend is predicted to continue as the technology continues to gain widespread acceptance and diversifies its application base. BLI is an optical analytical technique that measures interference patterns from white light reflected by two surfaces at the tip of a disposable biosensor: (1) a proprietary reference surface (constant) and (2) a sample or chemistry surface (variable). It monitors in real time the interaction between two different molecules with one—the ligand—immobilized onto the biosensor surface, while the other—the analyte—is kept in solution. Biosensors are coated with and are covalently or non-covalently linked with different biological molecules that allow for kinetics or quantitation measurements of the molecules bound to the tip [2,3].

Molecular interaction information, like kinetic rate constants, affinity binding constants, and specific molecule quantitation are some of the main characterization information that can be deducted from a BLI platform. Such information is necessary when characterizing and studying molecular interactions and is important for complementing other technologies when providing functional information of a molecule. In a typical BLI binding kinetics experiment, the assay begins with an initial baseline using an assay buffer, followed by a ligand molecule immobilization on the surface of the biosensor (loading), followed by another baseline step and association or analyte binding step, then finalized with dissociation in the presence of buffer only. The binding response is measured and reported in real time in the form of a sensorgram trace, which is then fitted using specified algorithms based on known binding models [2]. Kinetics measures how fast an interaction occurs and is depicted by the relationship between association rate constant (k_a_) and dissociation rate constant (k_d_). These constants are expressed as k_a_, M^−1^ s^−1^ and k_d_, s^−1^. Affinity measures how strong the interaction is and is depicted when binding reaches an equilibrium. It is usually referred as affinity constant (K_D_) or dissociation equilibrium constant, which is expressed in molar as the concentration of analyte required to occupy 50% of the surface ligand sites at equilibrium. These constants are readily calculated during software data analysis. For quantitation experiments, the molecule(s) bound to the biosensor are quantified in relation to a set of standards of similar characteristics. Software algorithms based on known curve equations deliver quantitation and CV% information [2,3].

Traditional assays, like equilibrium binding assays and endpoint assays, are cumbersome and fail to provide complete information about the interaction. Other traditional technologies, like enzyme-linked immunosorbent assays (ELISAs), high performance liquid chromatography (HPLC), native-PAGE gels, capillary electrophoresis, and single radial immunodiffusion (SRID) are encumbered by drawbacks that include long assay times, extensive hands-on labeling, and low throughput in some cases. In addition, techniques such as ELISA and SRID can exhibit high variability, resulting in lower accuracy and poor precision. Bio-Layer Interferometry (BLI) combines the high-throughput characteristics of a 96-well or 384-well plate format, with improved precision, reproducibility, and ease of use. For higher throughput, BLI technology can be combined with a robotic platform [4]. Other biosensor technologies like SPR require extensive instrument maintenance, buffer/sample treatments, and microfluidics maintenance that add time and cost of operations.

Accurate label free kinetics and quantitation of molecules is fundamental in vaccine studies as it allows scientists to better understand the mechanisms of the molecular interactions without the need to label molecules, which can be time consuming and may interfere with the molecules’ biological activity. The global vaccine market is ever more demanding as populations grow and microbe adaptability increases, thus there is pressing needs for technological advances to keep up with the demand. A growing number of vaccine manufacturers are opting to work with new, well-defined materials such as purified protein antigens isolated from a natural or recombinant vector, specific polysaccharides, oligosaccharide protein conjugates, and nucleic acid constructs. These advances and strategical diversities are well suited to work with the BLI biosensor technology due to the label free and real-time aspects of the technology and their incredibly high throughput and reliability.

## 2. BLI Applications on Vaccine Research and Development

BLI is an established technology that has already played a role in a number of vaccine studies. Because BLI technology can be used for kinetics and affinity determination as well as specific quantitation of molecules bound to the tip of the biosensors, it provides a vast array of applications and uses. BLI can assist in vaccine research and development in many ways, including epitope design, characterization and recognition studies, pathogen diversity and distribution, antibody affinity and development, host immune response characterization, diversity and distribution, nucleic acid and molecular pathogen-host interaction studies, and therapeutics and clinical studies. More applications are still to be discovered. At present, BLI technology has already contributed to a wide range of vaccine studies, and a short summary enlisting different diseases is presented on Table A1 (Appendix A).

The incredible diversity of microbes and their ability to adapt require constant investigation of the molecular interactions and modes of action of diseases. BLI technology has proven to work with a multitude of strategies involved in understanding the disease mechanism of action and possible modifications that allow for improved vaccine potency. The following examples show different applications and strategies employing BLI technology in the field of vaccine studies.

## 3. Epitope Design and Epitope Capture Approaches

Understanding the interaction between microbes and their host is a big step in improving or developing new vaccines. Epitope design and epitope capture approaches focus on the microbial molecular structure and how different epitopes may have different potency, functions, and targets that ultimately impact vaccines. The following examples show strategies that employ BLI and how they contribute to the understanding and characterization of epitope design toward vaccine development and improvements.

### 3.1. Site-Directed Mutagenesis of Epitopes

**Figure 1:**
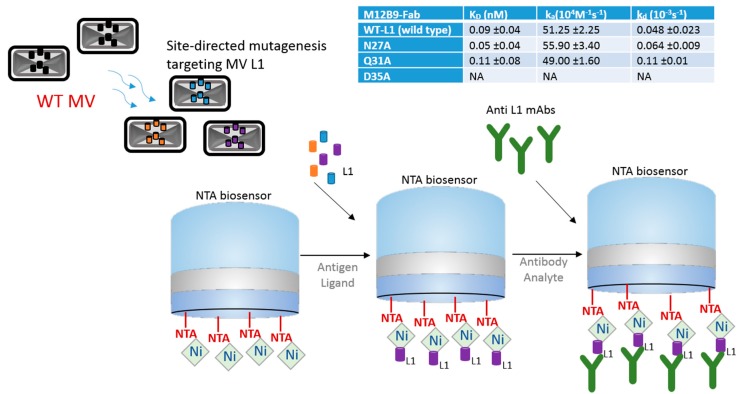
Schematics of bio-layer interferometry (BLI) strategy using a nickel-charged tris-nitrilotriacetic (NTA) biosensor for the identification of viral L1 residues that are most active during vaccinia infection. Site-directed mutagenesis was used to alter L1 residues, and BLI was used to test their affinity to anti-L1 mAbs. Table shows binding constant results of M12B9-Fab versus wild-type, N27A, Q31A, and D35A. K_D_ is affinity constant, k_a_ is association constant, and k_d_ is dissociation constant. Table data was derived from published data [6] with author consent.

Even though a vaccine against small pox is well established and was one of the first vaccines to be developed, orthologous strains are now a concern [5]. Since large-scale vaccination efforts against smallpox ended, the general population may no longer be protected against orthopoxviruses, which include monkeypox virus and various strains of cowpox viruses. BLI technology is being used as a tool to help in the search of cross-species neutralizers and to help with vaccine design and development against the vaccinia virus. A well-orchestrated BLI strategy is summarized and illustrated on Figure 1, in which the authors studied the L1 epitopes to understand the protective mechanism of the anti-L1 antibody, as the L1 antibody is an important target for viral neutralization [6]. L1 residues N27, Q31, and D35 were subjected to single-alanine substitution using site-directed mutagenesis, and the mutated recombinant L1 proteins were immobilized onto Ni-NTA biosensors via its C-terminal hexahistamine tag, and anti-L1 mAbs used as analyte. By measuring the affinity constant (K_D_), association rate constant (k_a_) and dissociation rate constant (k_d_) using 1:1 binding model, the authors compared the binding kinetics of WT-L1 (wild type), N27A, Q31A, and D35A against mAbs [6]. As a result, they found no binding of D35A against M12B9-Fabs, while no significant difference was observed between wild type and N27A and Q31A (Figure 1, table). No significant effect on binding affinity was observed with other L1 mAbs studied. These results show that M12B9 binds with L1 protein with high affinity, but a single D35A substitution in L1 abolishes the binding, which proves that D35 side chain is essential for binding a group of potent neutralizing antibodies.

This study shows how BLI can greatly contribute to the understanding of functional studies of proteins involved in viral recognition, which can lead to improved vaccine efficacy in the future.

In this case scenario, the epitope is immobilized to the biosensor first, and the mAb antibodies in solution are allowed to bind to the epitopes. This approach is useful for site-directed mutagenesis research because BLI assists in testing the modified peptides for their affinity and binding characteristics, providing real-time functional answers. Site-directed mutagenesis, gene knock-out or knock-in, and insertions and deletions are used for genetic manipulation. Recently, the understanding of the mechanisms involved in the clustered regularly interspaced short palindromic repeats (CRISPR-Cas) system has brought great advancements because it enables precise gene modifications [7]. Label-free biosensors are useful for bridging the gap between gene manipulation and biological function by providing more refined molecular characterization, such as quantitation data, kinetics, or affinity information of protein-protein interactions as well as RNA-protein or DNA-protein interactions.

### 3.2. Epitope Scaffolding

**Figure 2:**
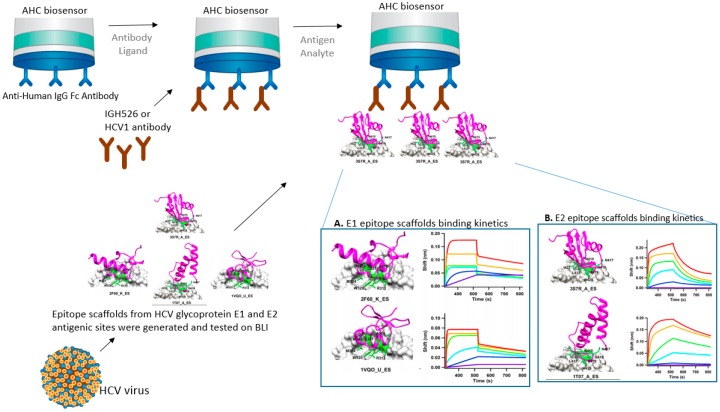
Schematics of BLI strategy using anti-human IgG Fc (AHC) biosensor for the characterization of Hepatitis C viral (HCV) epitope scaffolds (magenta) generated from antigenic sites of the HCV envelope glycoproteins E1 and E2. (**A**) Scaffold models and corresponding BLI sensorgrams showing fast on/fast off binding kinetics of E1 peptide and epitope scaffolds (2F60_K_ES and 1VQO_U_ES) to IGH526 antibodies; (**B**) Scaffold models and corresponding BLI sensorgrams showing fast on/slow off binding kinetics of E2 peptide and epitope scaffolds (3S7R_A_ES and 1T07_A_ES). All sensorgrams and epitope scaffolds reprinted from He et al. 2015 [10] with author permission.

Epitope scaffolding makes use of computational design for grafting of an epitope of interest onto a heterologous protein scaffold [8,9]. This epitope-focused vaccine design strategy can be very successful in improving vaccine potency or finding an effective vaccine target. In the following example, BLI was used to test HCV epitope scaffolds’ affinity to neutralizing antibodies. Contrary to smallpox, there are no vaccines against hepatitis C virus (HCV). The development of a prophylactic vaccine against HCV has been hindered due to the great diversity in the viral structure and host immune response. He et al. [10] designed HCV epitope scaffolds from the antigenic sites of glycoproteins E1 and E2. The authors searched and filtered potential scaffolds using a matrix meta-server method composed of six different databases: TM-align(F), TM-align(C), SPalign, CLICK, FAST, and Mammoth. Then, the authors used MD simulations to study the dynamics of potential epitope scaffolds in solution. The selected epitope scaffolds were his-tagged, transiently expressed in cells, purified, and their affinities tested on an BLI Octet instrument. Such multi-scale scaffolding is promising for such complex pathogen-host interactions. Structural modeling and computational designs can be tested experimentally for activity and affinity using the different platforms of BLI, transforming theory into reality. In their research, the authors used a BLI instrument—an Octet RED96—to show binding affinity between HCV epitope scaffolds to the neutralizing antibodies, which helped to characterize and select most efficient scaffolds (Figure 2) [10].

In this case scenario, the antibodies are bound to a preexisting IgG on the surface of the biosensor and the epitope scaffolds in solution are allowed to bind to the secondary antibody. The use of label-free biosensors like BLI and SPR for the biochemical characterization of epitope scaffolds has contributed to several case studies reported for influenza [11], RSV [12], and HIV [13]. Biosensor technologies provide affinity and kinetics binding information that is crucial for characterization studies. Like SPR, BLI can provide the on and off rate constants of antibody binding to the antigen by fitting a model function to the binding data, therefore providing the affinity of the interaction.

### 3.3. Epitope Binning

**Figure 3:**
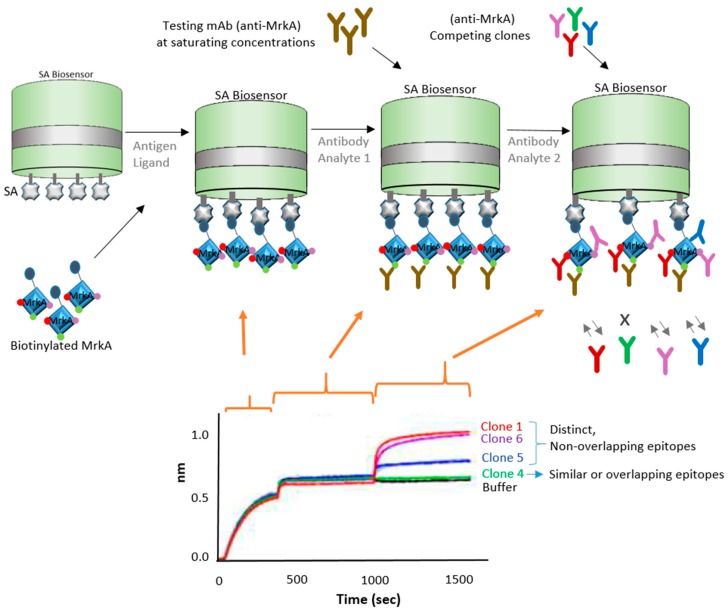
Schematic drawing showing in-tandem epitope binning strategy using a biofilm formation protein from *Klebsiella pneumoniae*. Diagram depicts biotinylated MrkA antigen immobilized on a streptavidin (SA) biosensor and exposed to competing antibody clones, which interact with corresponding epitopes. Sensorgram, adapted from Wang et al. 2017 [14] with permission, shows binding curve of different clones; buffer and clone 4 (binding site not available) are controls.

Epitope binning has been used for a number of years as a way to characterize and sort a library of antibodies into bins that bind distinct epitopes on the specific antigen. In this next strategy, BLI is demonstrated to be an ideal tool to expedite epitope binning toward vaccine development. In this case, the focus of study was antibiotic-resistant bacteria, which have been causing increasing public health concerns. Wang et al. [14] explored an antibody-based therapy for possible protection against *Klebsiella pneumoniae* bacteria, which is known to cause nosocomial infections. They focused on the protein antigen MrkA, which is involved in biofilm formation and early onset infection. In their study, the authors screened and characterized epitopes by panning single-chain variable fragment (scFv) antibody phage libraries against the recombinant MrkA protein. They started with more than 4000 colonies and narrowed down to four clones by utilizing a series of methodologies: (a) binding specificity via dual expression vector pSplice.V5; (b) followed by bacterial scFv.Fc expression and Opsonophagocytic killing (OPK) in vivo activity assessment; (c) scFv sequencing, (d) BLI epitope binning, and final screening for clone assessment they did binding ELISA, OPK affinity assays, and BLI epitope binning. The resulting four clones were further characterized using BLI technology. For the characterization of the four remaining clones, the authors used a biotinylated antigen captured on the BLI streptavidin biosensors and allowed the protein to bind to an antibody bin. Two of the remaining clones showed similar binding profile as a previously characterized KP3 antibody. Figure 3 shows a diagram of one of these clones and the BLI approach used.

This strategy shows use for finding anti-MrkA antibodies that have strong affinity for the MrkA epitope. BLI Octet instruments are accepted as a complimentary tool for epitope binning because of their versatility as they enable the parallel analysis of 96 independent analyte/ligand pairs [15]. Biosensor platforms can assist with different types of binning assay formats—in-tandem, premix, and classical sandwich epitope binning—due to their label-free, real-time capabilities. Usually, classical sandwich and in-tandem formats are used for crude monoclonal antibody (mAb) supernatants, while premix assays are used with purified mAbs for quantitation assays [16,17]. BLI works with all the binning formats. For crude samples, SPR and SPRi technologies are not ideal because the more complex samples can cause clogs and blockages on the microfluidics systems [18]. BLI also offers many advantages over traditional technologies such as ELISA for epitope binning because it provides real-time information and label-free experimental design. ELISA, on the other hand, may distort molecule conformation, miss information on low affinity antibody pairs, and limit the ability to troubleshoot the assay due to its limited end-point, label-dependent technology [19]. In the example described above, ELISA is useful as a complementary technology and can be used in combination with BLI and other technologies. Although difficult to use for in-tandem assays, ELISA is traditionally used for sandwich and premix assays.

BLI-delivered epitope clustering using the in-tandem strategy was developed for therapeutic antibody discovery to study a glucose-dependent insulinotropic polypeptide (GIP) receptor with the purpose of possible diabetes and obesity treatment [20]. The flexibility of BLI is useful for a variety of epitope studies and mAb selection.

For primary screening of large libraries of unpurified mAbs expressed in crude conditioned media, kinetics screening using RT-LF biosensor technologies provide rapid selection of potential candidates delivering rapid screening of more than 2000 mAb clones. BLI Octet technology, such as the HTX, can reliably measure kinetics of mAb-antigen interactions for a wide range of binding affinities. Data generated using titration kinetics (TK) and single cycle kinetics (SCK) assays were comparable when using BLI HTX, Biacore 4000 (SPR), and Mass-1 (SPRi) biosensors [21]. Greater advantage of BLI over other RT-LF biosensor technologies include reduced hands-on time, reduced assay time, and higher throughput, as well as sample recovery after the end of the experiment [21].

## 4. Antibody Design and Antibody Capture Strategies

### 4.1. High-Throughput Antibody Screening

**Figure 4:**
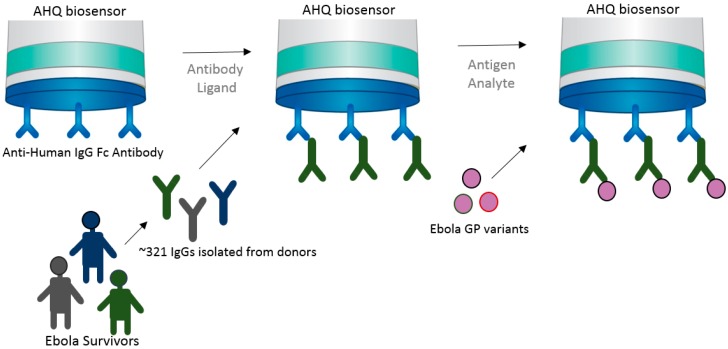
Schematic drawing showing the high-throughput strategy used for screening and mapping antibodies in relation to corresponding Ebola viral GP antigen variants. In this case, an anti-human IgG Fc antibody that coats the AHQ biosensor was used to capture the antibodies from Ebola survivors [24].

High-throughput capability is very important for vaccine development in many ways. From epitope screening to antibody screening, the ability to test a multitude of targets to funnel toward the few molecules that provide highest potency is a crucial aspect of immunotherapeutics and vaccine development. Immunotherapeutic ZMapp^TM^ is currently used for EBOV treatment, which targets the Ebola virus surface glycoprotein (EBOV GP) [22,23]. However, very little is known about the actual interaction of mAbs and the virus. This understanding is crucial for the future development of a potent and effective Ebola vaccine. In an attempt to better understand the biological interaction between virus and host, the Fortebio Octet HTX system, in conjunction with an anti-human IgG Fc (AHQ) biosensor, was used to map the antigenic binding specificities between GP (glycoprotein) variants against a population of over 300 GP-specific mAbs (monoclonal antibodies) isolated from a donor survivor to the Zaire outbreak (Figure 4) [24]. This initial high-throughput screening was important in narrowing down a potent target. Bornholdt et al. [24] were able to identify a mAb targeting the GP1/GP2 interface and GP stalk region, demonstrating great progress in the identification of mAbs for the next generation of EBOV immunotherapeutics.

The approach used in this case takes advantage of a preexisting antihuman IgG bound to the AHQ biosensor to immobilize the human IgGs in large quantities and bind to them to the epitope variants in solution. This approach shows the versatility of BLI in delivering fast and high-throughput results, allowing one to rapidly make kinetics analysis and bin neutralizing mAbs into competition groups in order to build potent immunotherapeutics.

The reliability and high-throughput of BLI platforms can assist in a multitude of vaccine research and immunotherapeutics applications. Recently, Li et al. [25] found its use for serologic surveys and seroepidemiologic investigations for the identification of influenza hemagglutinin (HA) subtypes specific antibody responses. The influenza A virus HA glycoprotein is important during initial stages of infection, in which it is responsible for binding the virus to sialic acid present on the host cell membrane [26]. The serosurveys utilize the HA protein to evaluate and estimate the potential disease severity. Traditional serological surveys are performed by hemagglutination inhibition (HI) and virus microneutralization (MN) assays [27,28]. When evaluating potential new technologies, Li et al. [25] found that BLI presents high sensitivity and expected specificity for human sera from patients exposed or unexposed to heterologous subtype influenza HA.

### 4.2. Bispecific Antibody Capture

**Figure 5:**
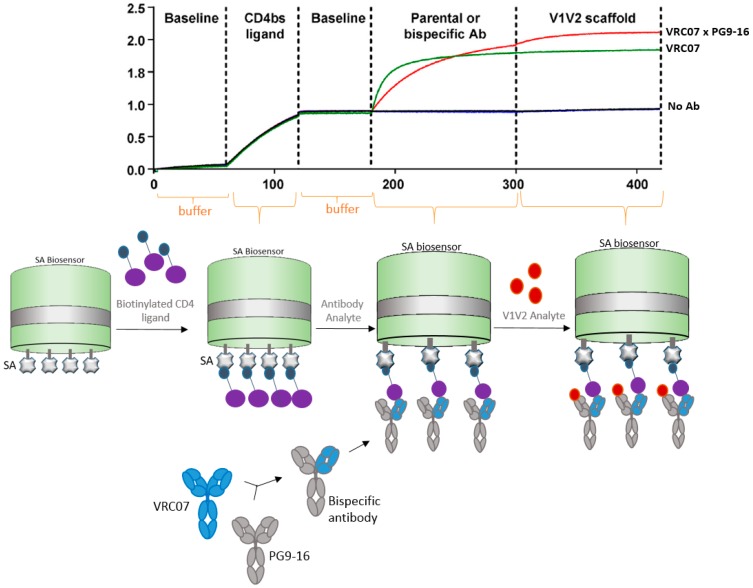
Diagram showing how BLI can be used for characterization of bispecific antibodies. In this example, bi-specific antibodies were generated to harbor Fc regions of both VRC07 and PG9-16 neutralizing arms against HIV virus. The VRC07 arm was bound to the streptavidin (SA) biosensor containing its target CD4 antigen, then the second arm PG9-16 allowed to bind to its V_1_V_2_ antigen, in a classic sandwich approach. Sensorgram, reprinted with permission [29], shows VRC07 + PG9-16 bispecific antibody (red line) versus VRC07 (green line) and no antibody (Ab) (dark blue line) control binding profiles.

Antibodies can be designed and manipulated to serve a variety of applications in medicine and science. In the past few decades, bispecific antibodies have greatly contributed to diagnostic and therapeutic industries, including vaccine research and development. Corroborating with advances in bispecific antibody research, BLI technology has proven to assist in the characterization of bispecific antibodies. In this next example, Asokan et al. [29] characterized bispecific antibodies targeting different epitopes of the HIV1 envelope protein. The authors developed four different bispecific antibodies combinations, which demonstrated various levels of neutralization. Increased broad and potent neutralization was observed with a bispecific antibody containing arms that bind to the different regions of HIV virus and the CD4 and V_1_V_2_ antigens (Figure 5).

In this case scenario, one arm of the bi-specific antibody was captured by the antigen that was bound to the SA biosensor. The second arm of the bispecific antibody bound to the second antigen. This way the bi-specific antibody has augmented neutralizing abilities because it recognizes two different sites of the HIV virus.

## 5. Virus Capture Strategies

### 5.1. VLP (Virus-Like Particles) Capture

**Figure 6:**
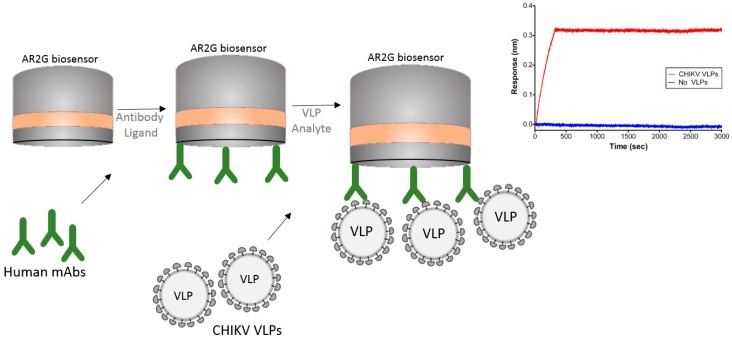
Schematic drawing showing a BLI strategy to capture virus-like particles (VLPs). mAbs were captured on the surface of the AR2G biosensor and the Chikungunya (CHIKV) VLPs allowed to bind to the mAbs. Sensorgram shows the binding of CHIKV VLP to the biosensor in comparison to the buffer control. Note that sensorgram was reprinted with author permission [30].

Another interesting example of the usage of BLI is its use with virus-like particles (VLPs). The Chikungunya virus (CHIKV) is an alphavirus that causes debilitating arthritic disease infecting millions of people worldwide. Studies are underway to develop a vaccine to improve therapeutic efficacy against this virus. Selvarajah et al. [30] performed experiments using VLPs to isolate a panel of human mAbs. The authors used an Octet RED system with an amine reactive biosensor (AR2G) to show binding specificity of one mAb (IM-CKV063) to the immobilized viral envelope glycoproteins E2 and E1. For these studies the authors used human mAb against CHIKV immobilized on an AR2G biosensor and then used purified CHIKV VLPs to bind to the mAbs (Figure 6). The ability of BLI technology to work with large particles like VLPs can greatly contribute to the study of vaccines.

In this strategy, the authors were able to capture the entire chikungunya VLP on the biosensor tip and study its interaction with mAbs.

### 5.2. Whole Virus Capture

**Figure 7:**
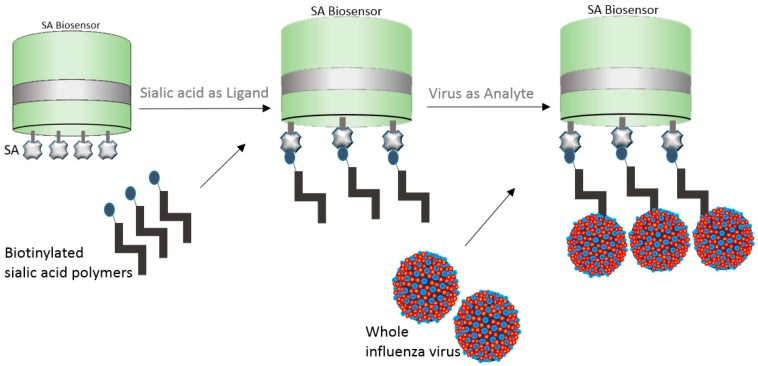
Pictorial representation of whole influenza virus binding to streptavidin (SA) biosensor by means of biotinylated sialic acid polymers.

In a brilliant example, whole influenza virus was captured onto the streptavidin (SA) biosensor by means of biotinylated sialic acid [31]. In an attempt to better understand human H7N9 and avian H7N3 virus diversity and receptor binding interactions, the authors bound biotinylated sialyl lactosamine polymers to SA biosensors and allowed the viruses to bind to the polymers (Figure 7). The authors calculated the relative amount of virus bound to the biosensor at different sugar loadings. The normalized virus binding response curves were in relation to the fractional saturation of the sensor surface and data fitting was improved by a variant of the Hill equation. The fractional saturation of the sensor surface (ƒ) was then related to the apparent equilibrium of the dissociation constant for virus binding. The K_d(virus)_ was then calculated as a function of the relative sugar loading for ƒ values ranging 0.05 to 0.95 [31].

The dip-and-read capability of BLI tends to favor whole virus and VLP applications over SPR instrumentations because BLI is not prone to some SPR-specific limitations. Some of the SPR specific-limitations include mass-transport limitations and artifacts caused by chemical microheterogeneity of the surface of the biosensor [32]. While usually compared to SPR technology, BLI has intrinsic characteristics that tend to favor multiple applications, which is mostly due to the ability to dip the disposable biosensor onto a solution containing samples and reagents, which tends to prevent artifacts from depositing on the biosensor and prevents fluidics limitations.

Immobilization of viruses and VLPs for real-time, label-free kinetics and quantitative characterization unlocks many potentials for vaccine studies.

## 6. Nucleic Acid Capture

**Figure 8:**
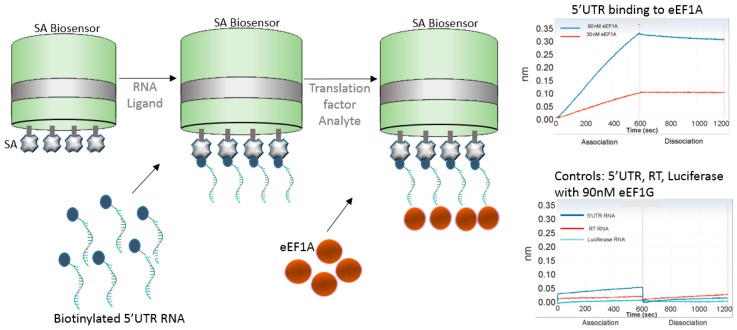
Schematics of BLI approach using SA biosensor to bind to biotinylated 5′UTR RNA and allowed to bind to translational factor eEF1A, during HIV-host interaction. Sensorgrams shows the binding of 5′UTR to the eEF1A factor versus the controls, reprinted with author permission [33].

The use of nucleic acids in vaccine development strategies have only grown in the past few years as novel approaches and proven efficacy continue to flourish. The next example shows the use of genomic RNA binding affinity to the eukaryotic translation elongation factor 1A (eEF1A), which is involved in the replication of HIV-1. Li et al. [33] used biotinylated 5′UTR, RT, and luciferase RNAs individually to test their binding affinity to eEF1A (Figure 8). The authors found that 5′UTR RNA strongly bound with eEF1A, while RT RNA showed week binding, and luciferase RNA had no binding. Similarly, no binding was shown when the authors used eEF1G translational factor, indicating specificity of 5′UTR RNA to eEF1A.

This approach shows use of BLI for studies involving nucleic acids and is quite promising for the understanding of viral/host interaction during vaccine development. BLI platforms are attractive for such application because of the lower sample volume requirements, non-destructive, non-dilution nature of the assays, and biosensor tips can be regenerated for the most part, allowing multi-use of the biosensor tip.

For nucleic acids applications, BLI has the added advantage that it can detect mechanistic structural nucleic acids changes upon binding. For example, Bruno [34] noticed that when they used long (200 base pair) aptamers for binding to peptide based biomarkers, the aptamer contracted, resulting into a smaller molecule that exhibited negative response signals upon binding to the peptide. Friedman et al. [35] found BLI to be invaluable for the characterization of aptamer pool enrichment, which is traditionally accessed by radio-labeling aptamers with either ATP [α^− 32^P] for in vitro transcription or ATP [γ^− 32^P] for 5′ end labeling. The authors found that radiolabeling disrupted aptamer function, while the RT-LF BLI biosensor technology provided fast high-throughput and reliable affinity information that could be tested in parallel for comparison of samples from different selection rounds [35]. In addition, radiolabeling is a cumbersome technique that requires careful manipulation and extra waste disposal costs. Aptamers are useful for vaccine and immunotherapeutic initiatives because highly specific aptamers can bind to viral proteins to inhibit or block fusion, penetration, or replication [34,36].

These examples clearly demonstrate the importance of understanding binding affinity, mAb specificity, host response, epitope specificity, and screening toward the development of a vaccine. Thus, the surge of BLI as an emerging method in the field of vaccine and immunotherapeutics that attracts labs worldwide and summons new application possibilities.

## 7. Conclusions

BLI technology has greatly assisted with vaccine research and development in recent years. With the various label-free BLI platforms and biosensors, it is possible to obtain detailed information from binding kinetics, specificity/affinity, immune responses, epitope mapping, and vaccine titer and concentration. This makes BLI a very powerful technique to be adopted in any laboratory involved in the development and production of vaccines and where scientists are trying to prevent or remediate pathogenic attack for the ultimate goal of protecting the population.

## Appendix A

**Table A1:** Citations using BLI technology toward vaccine research and development. Sources are peer-reviewed publications identified through Science Direct and Google Scholar.

Disease	Organism	Citation n#	Selected Reference
HIV	Lentivirus, virus	53	[29]
Flu/Influenza	Orthomyxoviridae, virus	36	[25]
Ebola virus	Filoviridae, virus	8	[24]
Dengue	Flavivirus, virus	6	[37]
Smallpox, variola	Vaccinia virus (VACV) orthopoxvirus, virus	6	[6]
Staph infection	*Staphylococcus aureus*, bacteria	5	[38]
Malaria	*Plasmodium falciparum*, protozoa	4	[39]
Chikungunya infection	Chikungunya (CHIKV), virus	3	[30]
Tuberculosis	*Mycobacterium tuberculosis*, bacteria	2	[40]
Middle East Respiratory syndrome	MERS-CoV coronavirus, virus	2	[41]
Hepatitis C (HCV)	Hepacivirus, virus	2	
Anthrax toxin	Bacillus anthracis, bacteria	2	[42]
Zika	Flavivirus, virus	2	[43]
Herpes	Herpesviridae, virus	2	[44]
Respiratory infection	Syncytial virus (RSV), virus	1	[45]
Hand, foot and mouth disease (HFMD)	Enterovirus 71 (EV71), virus	1	[46]
HMPV	Metapneumovirus, virus	1	[47]
Schistosomiasis	Schistosoma japonicum, Trematoda worm	1	[48]
CMV infection, congenital infections	cytomegalovirus (HCMV), virus	1	[49]
Whooping cough	*Bordetella pertussis*, *bacillus*	1	[50]
Clostridium difficile infection (CDI)	*Clostridium difficile*, *bacteria*	1	[51]
Marburg virus disease	*Marburg filoviridae*, *virus*	1	[52]
Diphtheria	*Corynebacterium diphtheria*, *bacteria*	1	[53]
Gastroenteritis, urinary infections, neonatal meningitis	*Escherichia coli*, bacteria	1	[54]
Listeriosis	*Listeria monocytogenes*, bacteria	1	[55]
Lung infection, opportunistic pathogen	*Pseudomonas aeruginosa*, *bacteria*	1	[56]
Otitis, pulmonary infections	*Moraxella catarrhalis*, *bacteria*	1	[57]
Vaginitis	*Trichomonas vaginalis*, protozoan	1	[58]
Chlamydia infection	*Chlamydia trachomatis*, *bacteria*	1	[59]
Pulmonary, urinary infections	*Klebsiella pneumoniae*, bacteria	1	[14]
Encephalitis	Japanese encephalitis virus (JEV), virus	1	[60]
